# Then, Now, Next: Unpacking the Shifting Trajectory of Social Determinants of Health

**DOI:** 10.3390/ijerph22101541

**Published:** 2025-10-09

**Authors:** Sherrie Flynt Wallington, Calistine Feger

**Affiliations:** 1 School of Nursing, George Washington University, Ashburn, VA 20147, USA; 2 Milken Institute School of Public Health, George Washington University, Washington, DC 20037, USA; cfeger@gwmail.gwu.edu; 3 GW Cancer Center, George Washington University, Washington, DC 20037, USA

**Keywords:** health equity, structural determinants, policy change, experiential education, social dynamics

## Abstract

This paper examines the evolving trajectory of the Social Determinants of Health (SDOH), tracing their development from early observational studies to contemporary, interdisciplinary frameworks that emphasize structural inequities and relational dynamics. It explores foundational milestones such as the Whitehall studies, the Heckler Report, and the World Health Organization’s conceptual models, which positioned SDOH as key drivers of population health. The paper highlights how upstream determinants—such as governance, policy, and socioeconomic systems—influence downstream health outcomes through mechanisms of social stratification and unequal access to resources. While SDOH are increasingly applied in clinical and educational settings, significant challenges persist, including underinvestment in community systems, fragmented care models, and political rollbacks of equity-centered policies. The paper critiques deterministic and deficit-focused framings of SDOH and underscores a shift toward more relational, context-sensitive, and agency-oriented approaches, reflected in the emerging concept of “social dynamics of health.” It highlights the importance of experiential education, competency-based curricula, and digital innovations in driving systemic transformation. Emphasis is placed on reimagining SDOH pedagogy and expanding interdisciplinary, data-driven research to bridge the gap between knowledge and practice. Amid shifting political landscapes, sustaining health equity efforts requires embracing adaptive, participatory models that acknowledge power, community agency, and structural change.

## 1. Early Underpinnings

The trajectory of understanding and addressing social determinants of health (SDOH) has evolved significantly over time, moving from early observations of the link between social conditions and health to a comprehensive framework influencing policy and action. As humans have evolved, how disease affects individuals cannot be attributed to a singular cause. For decades, healthcare and public health professionals have identified a correlation between negative health outcomes among disadvantaged, marginalized, and low-income populations [1]. These disparities are pervasive, often unjust, and deeply rooted in structural inequalities such as poverty and systemic discrimination, which are among the primary drivers of health inequities [2,3].

However, efforts to address these health disparities did not commence until 1967 with the United Kingdom (UK) Whitehall study, which demonstrated a health gradient based on social status; higher socioeconomic status (SES) predicts better health, while lower SES predicts poorer health [1]. A continuation of the Whitehall study in 1985 found that self-perceived health status and symptoms were worse in study participants in lower-status jobs [4]. This reinforced the concept of a social gradient, which is not limited to the most disadvantaged but extends across all levels of society, highlighting that even incremental differences in SES can lead to significant differences in health outcomes [5].

The preceding studies conducted by the UK revealed the field of study, health equity, to the rest of the world. As a result, Margaret Heckler, Secretary of the United States Department of Health and Human Services conceived a Task Force on Black and Minority Health in 1985 and submitted the first consolidated American report on minority health [1]. This report unveiled a perpetuating disparity in the burden of deaths and illnesses experienced by the Black community and other minority Americans as compared to the nation’s population as a whole [1]. These findings marked the first formal acknowledgment by the U.S. government of systemic health disparities tied to race and ethnicity, serving as a catalyst for ongoing efforts to improve health equity [3].

In 1999, Michael Marmot and Richard Wilkinson published a book entitled Social Determinants of Health, which presented the following claims backed by scientific evidence: “differences in health between population groups are due to characteristics in society, not differences in health care; when people change social and cultural environments, their disease risks change; the health gradient is not a matter of selection… health does not determine social positions, rather, social positions determine health” [6]. These claims aligned with a growing body of literature affirming that societal factors such as access to quality education, employment opportunities, and discrimination significantly shape health risks and outcomes [3].

In 2005, Marmot led the World Health Organization (WHO) Commission on Social Determinants of Health, which discussed the social gradient and the various ways in which material disadvantage combines with the effects of insecurity, anxiety, and lack of social integration to affect the health of those at progressively lower levels of SES [7]. In 2010, the WHO created the “Conceptual Framework for Action on the Social Determinants of Health”, which displayed the complex intersection of various factors and their influence on health outcomes [8]. The framework employs an analogy of a “waterfall” where “upstream” factors lead to “downstream” effects [8]. “Upstream” determinants (e.g., governance, macroeconomic policies, social policies, public policies, and cultural values) shape social hierarchies (e.g., power, prestige, and discrimination) [8].

These hierarchies, in turn, influence an individual’s socioeconomic position (e.g., social class, gender, ethnicity, education, occupation, and income) [8]. This position then affects “downstream” factors, like living conditions and access to healthcare, which ultimately impact health equity and well-being [8]. This model reflects the realities of structural determinants of health inequities, which generate and reinforce social class disparities through mechanisms such as housing segregation, education inequity, and labor market discrimination [3].

The framework emphasizes that addressing governance and policies is imperative to improving population health and promoting health equity. The combined efforts of health equity advocates have led the WHO to publicize the official definition of the social determinants of health as the non-medical factors that influence health outcomes including the conditions in which people are born, grow, work, live, and age, and the wider set of forces and systems shaping conditions of daily life [9]. These forces and systems include economic policies and systems, development agendas, social norms, social policies, and political systems [9]. Furthermore, understanding and acting on the SDOH is critical for ensuring that every individual has a fair and just opportunity to achieve optimal health regardless of their social circumstances [10].

## 2. Social Determinants of Health in Practice

There are numerous benefits to applying the SDOH in one’s practice. Applying an SDOH perspective when treating patients allows providers to better understand the broader social factors impacting a patient’s health, enabling them to identify potential barriers to care, provide more tailored treatment plans, and ultimately improve health outcomes, particularly for vulnerable populations [11]. For example, JAMA Pediatrics published a 2016 randomized clinical trial that discovered when pediatric providers screened families for social needs followed by connection to services, families reported social needs significantly decreased and child health outcomes ultimately improved [12]. This approach is particularly important in the U.S. context, marked by stark income inequality and racial disparities, where health outcomes are shaped more by social conditions than by medical interventions alone [13].

An SDOH approach can lead to improved patient engagement, reduced health disparities, and more effective interventions by addressing underlying social needs that may be contributing to health issues [11]. Research shows that addressing SDOH not only benefits individual patients but also supports broader societal goals, including economic stability, national security, and intergenerational well-being [14].

One of the primary ways healthcare providers are expanding beyond their traditional medical service delivery role to address the SDOH is by screening patients for social concerns and referring them to community services that can address identified needs [15]. However, there are many barriers to implementing SDOH screening programs and initiatives within the healthcare sector [15]. For example, some clinicians argue that SDOH screening may be ineffective or unethical if linkages to services are unavailable, while others question who should conduct screenings and what training should be required due to the lack of standardized guidelines regarding the SDOH [15]. Moreover, structural challenges such as underinvestment in community services, lack of integrated care models, and limited policy support have hindered the scale-up of effective SDOH interventions in clinical settings [16].

Despite these barriers, the health system remains a critical point of contact for vulnerable individuals and a powerful lever for addressing inequities. Interventions that combine clinical care with community partnerships have demonstrated success in mitigating social risks, reducing preventable hospitalizations, and disparities in chronic disease management [17]. For instance, the Accountable Health Communities Model, tested by Centers for Medicare & Medicaid Services (CMS), showed that systematic screening for housing, food, and transportation needs linked to service navigation significantly improved patient outcomes [18,19]. Therefore, the integration of SDOH into routine clinical care must be supported through education, policy, and cross-sector collaboration to realize its full potential in advancing health equity.

## 3. Theories, Models, and Frameworks

**Figure 1:**
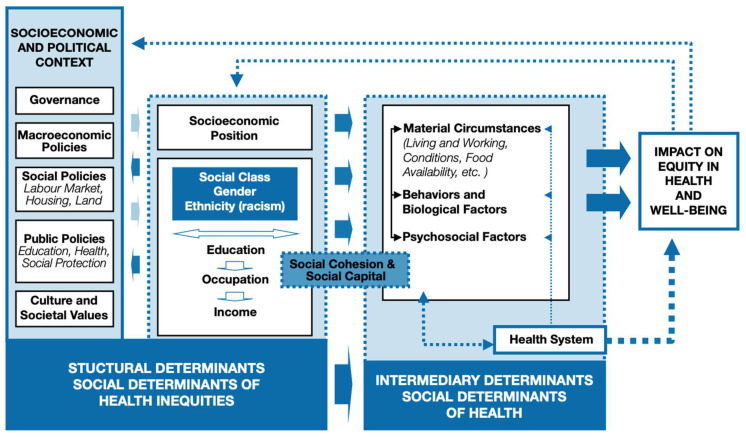
CSDH conceptual framework. Source: WHO [8]. A Conceptual Framework for Action on the Social Determinants of Health.

Understanding health inequities through the lens of Social Determinants of Health (SDOH) requires a comprehensive theoretical and conceptual grounding. The World Health Organization (WHO) defined SDOH as the conditions in which people are born, grow, live, work, and age, shaped by wider social forces and systems [20]. To conceptualize these dynamics, the WHO’s Commission on Social Determinants of Health developed a framework in 2010 that differentiates between structural and intermediary determinants of health [8]. Structural determinants include socioeconomic and political contexts, such as governance, policies, and societal norms, which shape social stratification and determine individuals’ socioeconomic positions—through factors like income, education, occupation, gender, and race. Intermediate (social) determinants are the specific conditions in which people are born, grow, live, work, and age. They are the direct results of the structural determinants and more directly impact health outcomes [8]. These positions, in turn, influence exposure to health risks and access to resources, contributing to health outcomes (see Figure 1).

The framework integrates three core theoretical perspectives: psychosocial approaches, the political economy of health, and eco-social frameworks [8]. These perspectives collectively explain causation through mechanisms like social selection (a selective force that occurs when individuals change their own social behaviors in response to signals from conspecifics, thereby influencing others’ fitness [21]), social causation (the idea that an individual’s social environment and circumstances can influence their health and well-being [22]), and life course perspectives (which examine how health develops across the lifespan shaped by the interplay of biological, social, and historical factors over time [23]). A key contribution is Diderichsen’s model, which illustrates how social context leads to stratification, unequal exposure, and unequal consequences of illness. Central to this framework is the role of power—not just as domination, but as a vehicle for agency and rights-based advocacy. This reframing positions health equity as inherently political, necessitating structural change and collective empowerment.

**Figure 2:**
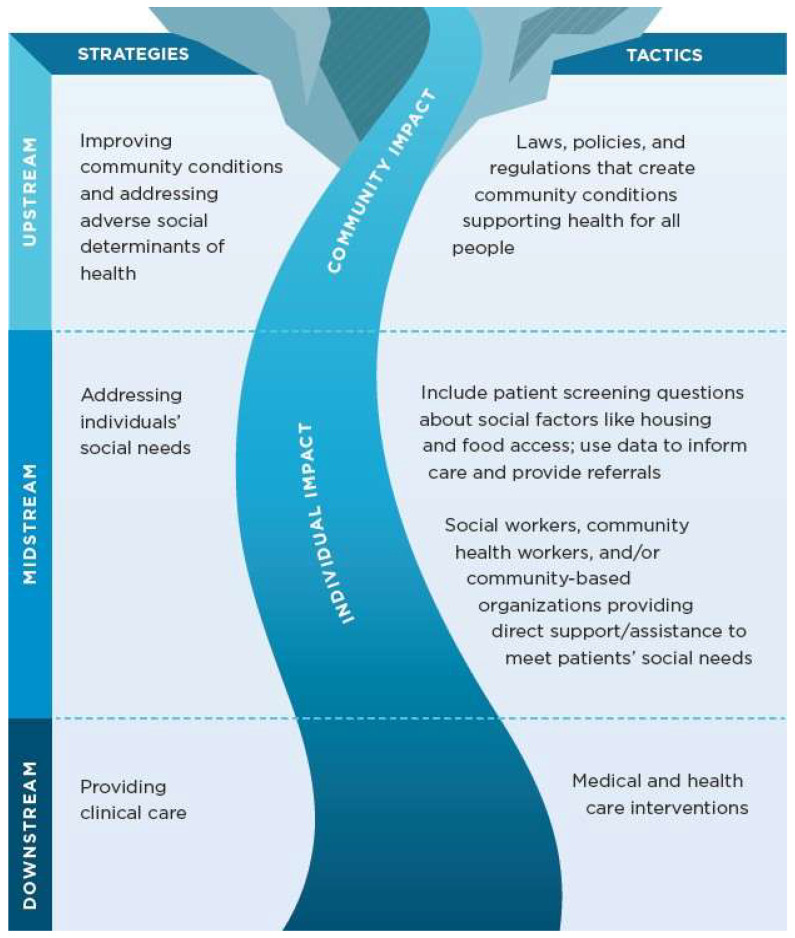
Social Determinants of Health and Social Needs Model. Source: Castrucci and Auerbach [24].

In parallel, Castrucci and Auerbach [24] introduced the Social Determinants of Health and Social Needs Model to articulate a tiered approach: upstream (structural inequities like poverty and discrimination), midstream (individual-level social needs such as food or housing insecurity), and downstream (clinical interventions and chronic disease management). Most nurses work at midstream and downstream levels, but upstream engagement through advocacy and policy reform is essential for sustainable health equity (see Figure 2).

Despite these conceptual advances, implementation challenges persist. In addition to the difficulties that providers face when trying to integrate the SDOH framework into clinical practice, public health professionals have critiqued the framework for failing to close the “health gap” across social classes [25]. In 2019, a convening of scholars highlighted the key limitations of traditional SDOH models. One major concern was the lack of attention to “relationality”—that is, how unequal distributions of power and privilege shape access to health-promoting resources. For example, a 2025 retrospective cohort study examined millions of hospitalized adults in New York and California, using a community-level SDOH index encompassing SES, education, neighborhood factors, food access, social context, and healthcare. After adjusting for demographic and clinical risk factors, the study found that patients from communities with weaker SDOH profiles experienced significantly higher one-year post-discharge mortality rates as compared to those with stronger SDOH profiles [26]. This finding underscores the need to transcend deficit-oriented models, recognizing that individual health outcomes are deeply intertwined with upstream factors, and to adopt relational perspectives that situate individual health within the broader structural and social systems. Furthermore, SDOH thinking often centers on deprivation, inadvertently portraying populations as passive victims of structural inequities and risk carriers, rather than as active agents capable of mobilizing social resources to advance health. This narrow and sometimes paternalistic framing not only simplifies complex social processes but also impedes the development of practical, systemic solutions to address inequities [25]. For example, a 2025 experimental survey study found structural messaging increased support for systemic policy solutions, illustrating how traditional deprivation-based SDOH frameworks obscure communities’ demonstrated capacity to mobilize against health injustices [27].

In response, education frameworks have emerged to prepare healthcare professionals to address SDOH holistically. The National Academy of Medicine and the Committee on Educating Health Professionals developed a framework grounded in transformative learning, interdisciplinary collaboration, and lifelong learning [28]. Educational domains emphasize experiential strategies such as community engagement, applied learning, and reflective practice based on Kolb’s experiential learning cycle [29]. These methods aim to deepen understanding of SDOH through real-world complexity and patient-centered contexts.

Specifically in nursing, initiatives like the AACN’s competency-based education (CBE) model advocate for integrating SDOH across curricula. This model promotes learning outcomes that evolve in complexity, assessed through diverse strategies including self-reflection, simulations, and unfolding case studies [30]. Programs like Chamberlain University’s Social Determinants of Learning Framework and the CARRN framework for rural nursing also reflect a shift toward culturally responsive and equity-oriented education [31,32].

Furthermore, newer heuristic models like Guilamo-Ramos et al. [33] provide operational frameworks that guide practitioners through identifying inequities, contextualizing determinants, and implementing targeted interventions. These evolving models underscore the importance of connecting theory to practice in ways that empower both providers and communities.

## 4. Research Gaps and Stimulating Research

Significant gaps persist in Social Determinants of Health (SDOH) research, especially in evaluating how health interventions impact equity and cost-effectiveness. The complexity of linking structural determinants to individual outcomes remains underexplored due to limited theory-driven approaches [25,34]. The Agency for Healthcare Research and Quality (AHRQ) [34] identified key research gaps, including the effects of interventions on diverse populations, the role of health systems in addressing social needs, and the long-term sustainability of care models. Additional gaps involve insufficient integration of patient preferences, limited use of participatory research, and underutilization of digital health data. There is also a pressing need to produce timely, actionable findings for health equity stakeholders and to use theories that connect fragmented evidence into coherent insights [34]. Moreover, a critical language gap exists: the term “determinants” may downplay resilience and agency, limiting the appeal and adaptability of SDOH frameworks in clinical and policy contexts [35]. Addressing these gaps can transform research into more responsive, equitable health systems.

## 5. Shifting Language and Trajectory (Landscape)

The terminology surrounding social determinants of health (SDOH) is evolving, with alternatives like “social drivers,” “non-medical risk factors,” and “social dynamics” gaining traction. These shifts aim to counter the perception that SDOH are fixed and deterministic, instead emphasizing community agency and the potential for change [35,36]. For instance, the term “social dynamics” better reflects the fluid, relational, and context-dependent nature of health influences and encourages more adaptive, trust-based partnerships [36]. While some caution that new terms may weaken the global policy recognition of SDOH [35], others argue that reframing can stimulate transformational thinking and better align with the complexities of human flourishing [37]. Importantly, language shapes thought and policy. This is particularly salient as recent federal policies in the U.S. challenge terms associated with diversity, equity, and inclusion [38]. These directives, including the termination of DEI and equity-related initiatives, heighten scrutiny of language used in research and grant proposals. Terms explicitly referencing systemic inequities may face review or disqualification, underscoring the strategic importance of framing proposals with adaptable terminology such as “social drivers,” “non-medical risk factors,” or “social dynamics.” This shift illustrates how language is not merely descriptive but deeply political, reinforcing the need for precise, context-sensitive terms that advance health equity amid evolving policy landscapes. Thus, critically reflecting on language use is essential for sustaining impactful public health discourse and action.

It is also essential to identify which terminology should be employed in various research or clinical settings. The National Center of Community Health Centers (NACHC) promotes the usage of “social drivers” when addressing policies, systems, and structures that fuel racial inequities in areas that influence health (e.g., healthcare) [39]. NACHC’s rationale is that “determinants” can have a sense of finality depriving individuals of agency over their health while minimizing accountability among policymakers for the social and political decisions that perpetuate these inequities. Literature explicitly employing the term “social dynamics” in relation to health is sparse, yet adjacent literature offers valuable conceptual foundations that can inform the use of this term. For example, ecosocial theory argues that health is shaped by dynamic interactions between biology, society, history, and political economy, which provides a robust theoretical grounding for viewing health as emerging from dynamic social-biological interplay [40]. Accordingly, the term “social dynamics” may be particularly useful in contexts where adaptability, agency, and relational change are most salient. According to the Centers for Disease Control and Prevention (CDC), “non-medical risk factors” are non-clinical influences on health, well-being, and quality of life (e.g., economic, social and environmental factors) [41]. These non-medical risk factors include elements such as poverty, housing instability, education, and transportation barriers [41], aligning closely with broader SDOH frameworks but offering a term well-suited for clinical documentation, insurance, and public health reporting.

## 6. Evolving Framing and Political Dynamics of SDOH

Federal executive actions have profoundly shaped the landscape of the social determinants of health (SDOH) in recent years. Building on Dawes’ concept of the political determinants of health, these federal actions can be understood not only as policy reversals but as deliberate political choices that distribute power and resources in ways that sustain inequities. Political determinants operate as the instigators of social determinants, shaping the contexts in which housing, education, healthcare, and labor policies unfold [42,43]. This framing emphasizes that current federal rollbacks are not neutral administrative shifts, but structural reallocations of opportunity and disadvantage that directly cascade into population health outcomes. The Biden administration had advanced policies aimed at expanding access to healthcare, education, housing, and economic security—core dimensions of SDOH. However, the early months of President Trump’s second term have reversed many of these efforts through sweeping executive orders that dismantle diversity, equity, inclusion, and accessibility (DEIA) initiatives and alter federal commitments to health equity and prevention infrastructure [38,44].

This policy reversal marks a fundamental shift in the federal government’s approach to SDOH, with significant implications for health equity, institutional trust, and public health practice. Trump’s executive directives—including terminating all DEIA programs across federal agencies, banning gender-affirming care for youth, legally redefining ‘sex’ as strictly binary and immutable, thereby erasing recognition of gender identity and intersex variation, withdrawing from the World Health Organization (WHO), dismantling the Department of Education, and drastically reducing the federal workforce—signal a structural retraction from the policies and frameworks that previously supported upstream, equity-centered approaches to public health [44,45,46,47,48].

According to the World Health Organization [49], contemporary SDOH frameworks emphasize prevention, value-based care, integration of health and social services, and collaborative, data-driven approaches to reducing disparities. However, these foundational pillars are directly undermined by current federal actions.

For instance, the repeal of DEIA frameworks threatens to erode institutional awareness of and accountability for structural disadvantages that shape health disparities. DEIA programs serve as vital infrastructures that inform culturally competent care and promote community trust—an essential precondition for equitable health partnerships [36]. A systematic review of multiple empirical studies supports these claims by reporting that DEIA initiatives elevate patient stratification, enable patient-provider trust, promote greater treatment adherence, increase utilization of preventative care, and improve team performance and clinical retention [50]. Without these structures, the capacity of health systems to recognize and address intersectional vulnerabilities is diminished.

Integration of SDOH into healthcare—through screening, referrals, and intervention around issues like housing, food insecurity, and employment—depends on strong interagency collaboration and supportive federal policy. The dismantling of the Department of Education and the withdrawal from the WHO weaken the cross-sectoral partnerships that make such integration viable [44,47]. Moreover, value-based care models, which emphasize improved outcomes over service volume, rely on robust federal data infrastructure and alignment with health equity goals [49]. The rollback of equity initiatives, along with drastic cuts to the Department of Health and Human Services (HHS), pose a direct threat to these mechanisms [48].

In addition, the Trump administration’s redefinition of sex as binary and immutable and the prohibition of federal funding for gender-affirming care undermine inclusive and affirming health environments. These actions reverse protections that enabled LGBTQ+ populations—who experience pronounced health disparities—to access appropriate and respectful care [45,46]. Studies have proven that anti-LGBTQ+ policies such as the denial of gender-affirming care are linked to higher rates of depression, suicidal ideation, and delayed healthcare [51]. In addition, as Adler et al. [52] noted, addressing SDOH upstream is more cost-effective and impactful than intervening after disease has developed. Yet the current policy trajectory undermines this preventive orientation by cutting the very programs that facilitate upstream interventions.

Language shapes policy, and thus, reframing from SDOH to “social dynamics of health” provides a more flexible and empowering lens for addressing health inequities [36]. This dynamic framing acknowledges that community-led transformation and structural realignments are essential to achieving health equity. However, under the current administration’s policy direction, such community-engaged, transformational approaches face considerable barriers.

Further compounding these threats are actions targeting educational institutions and public discourse. The termination of funding for programs perceived to promote “gender ideology” or “anti-American” perspectives in K–12 education, combined with efforts to shutter the Department of Education, restricts the development of health literacy, civic engagement, and critical consciousness—skills crucial for navigating and transforming the social landscape of health [47]. Based on findings from several empirical studies, this policy action is likely to have a severe impact on population health. For example, a 2025 observational analysis that estimated life expectancy across 3110 U.S. counties (2000–2019), stratified by educational attainment, found that life expectancy was 11 years greater for the most educated as compared to the least educated groups [53]. Similarly, a 2024 global systematic study reported that educational attainment was associated with a 1.9% reduction in all-cause adult mortality [54]. Together, these findings highlight how undermining educational access directly promotes poor health outcomes and exacerbates health inequities.

As Elgarblog [55] emphasizes, the conditions that shape health are deeply political and economic. Addressing SDOH thus demands not only technical interventions but transformative public policy that challenges structural inequities. However, austerity-driven governance, combined with politicized attacks on reproductive, gender, and racial equity, restricts the scope of such transformative potential.

Therefore, the evolving political dynamics under Trump’s second term pose significant challenges to health equity. These executive orders systematically dismantle the federal scaffolding that supports social, educational, and healthcare systems responsive to community needs and structural inequities. In doing so, they disrupt the shift toward more relational, context-sensitive, and preventive public health frameworks.

## 7. Pedagogical Strategies in Teaching SDOH

Effective pedagogical strategies for teaching Social Determinants of Health (SDOH) are central to developing health professionals’ capability of advancing health equity through both clinical practice and structural advocacy. As recognition of the impact of SDOH on patient outcomes has grown, so too has the urgency to reimagine healthcare education in ways that engage learners with the real-world complexity of social and structural drivers of health.

A major shift in pedagogical focus has been the transition from didactic, lecture-based teaching toward active and experiential learning models. Experiential learning—rooted in Kolb’s experiential learning theory—emphasizes reflective observation and applied engagement, allowing learners to confront personal biases and assumptions in relation to health inequities [28,29]. Strategies such as community-based service learning, interdisciplinary case-based learning, simulation exercises, and reflective journaling are now seen as essential components of SDOH pedagogy [56,57].

Simulation-based education has emerged as a particularly powerful method for immersing students in the social realities their patients face. For instance, pharmacy and nursing education programs have employed poverty simulations, cultural role-plays, and standardized patient encounters to build empathy, cultural competence, and practical problem-solving skills [58]. These approaches are increasingly adopted across a variety of health professions, including medicine, public health, and allied health fields, to stimulate critical thinking and responsiveness to the SDOH in care delivery. These activities allow learners to grapple with barriers such as low health literacy, transportation challenges, and housing insecurity—factors often invisible in clinical settings yet critical to health outcomes. Such simulations bridge the gap between conceptual understanding and clinical application, increasing engagement and retention while creating low-risk environments for learners to practice communication and decision-making [59,60].

To support sustained skill development, leading institutions are integrating SDOH instruction longitudinally across curricula. Programs like the one at Arizona State University incorporate courses on social justice and health equity, using blogging, community assessments, and interdisciplinary discussions to deepen student understanding and commitment to social change [61]. Others, like the University of California Davis’s “road trip” immersion through underserved communities, use place-based learning to cultivate respect, cultural humility, and geographic awareness of health disparities [62].

National standards have increasingly aligned with this pedagogical transformation. The American Association of Colleges of Nursing (AACN) introduced competency-based education (CBE) frameworks that promote progressive mastery of SDOH-related skills through diverse assessments, including self-reflection, simulations, and community engagement [30,63]. These competencies span both undergraduate and graduate nursing education, aiming to prepare health professionals as transformational leaders in health equity.

Further, educational frameworks such as the Social Determinants of Learning model at Chamberlain University expand the focus beyond content delivery to creating inclusive environments that address learners’ own social needs. This includes promoting research on racism, structural discrimination, and systemic health disparities [31].

Despite these innovations, gaps remain. Many programs still underrepresent upstream determinants such as policy, governance, and systemic racism, focusing instead on midstream and downstream factors. Moreover, simulation-based strategies often overemphasize cultural competency at the expense of broader structural insights [58]. To address this, emerging heuristic models—such as Guilamo-Ramos et al.’s three-step framework—encourage educators to guide students in identifying inequities, contextualizing determinants, and designing targeted interventions that reflect real-world leverage points [33].

Ultimately, effective SDOH pedagogy requires more than curriculum reform; it demands a transformation in how health professionals are trained to see and act upon the social conditions shaping their patients’ lives. Embedding interdisciplinary collaboration, community partnerships, and continuous reflective practice into health education holds the promise of cultivating a future workforce equipped not only with clinical acumen but also with the vision and courage to advance health equity.

## 8. Stimulating Research on SDOH

Stimulating research on Social Determinants of Health (SDOH) is essential for addressing persistent health inequities and bridging the gap between knowledge and action. Recent advances in data infrastructure and digital innovation are transforming the SDOH research landscape, enabling more precise identification, analysis, and intervention design. Leveraging real-world data (RWD) and electronic health records (EHRs), researchers are now able to integrate contextual SDOH with individual-level social risks and needs to better predict health outcomes [64].

Emerging platforms such as the Sociome Data Commons and Health Equity Explorer exemplify this shift, offering harmonized geospatial datasets and scalable informatics tools to support community-level interventions and policy development [65,66]. Moreover, natural language processing (NLP) tools are increasingly utilized to extract complex social conditions—such as housing instability or incarceration—from unstructured clinical notes, thus expanding the scope and depth of SDOH research [67,68].

Equally important are implementation studies that translate these tools into practice. Innovative frameworks like the Social and Environmental Determinants of Health Informatics Maturity Model guide organizations in assessing and improving their readiness to integrate SDOH into care delivery [69]. These advancements are also prompting policy shifts. For instance, the Centers for Medicare and Medicaid Services have mandated SDOH data collection beginning in 2024, reinforcing the integration of social context into healthcare delivery [64].

Ultimately, this expanding body of research signals a paradigm shift—where informatics-driven inquiry, policy alignment, and interdisciplinary collaboration converge to address the structural roots of health disparities. Continued investment in SDOH research infrastructure will be vital to advancing equity-focused healthcare systems and learning communities.

## 9. Conclusions

In order for public health practitioners to effectively implement this call to action, research directives must be prioritized. Methodological priorities include advancing theory-driven approaches that link structural factors to outcomes across diverse populations, leveraging community engagement through participatory research models, reframing “social determinants” as “social drivers”, “social dynamics”, or “non-medical risk factors”, evaluating the long-term sustainability of care models, harnessing digital and data-driven innovations to better capture social risks, and generating actionable evidence on the equity and cost-effectiveness of upstream versus downstream interventions. This reframing, such as the adoption of “non-medical risk factors” in surveillance and reporting [41], bridges clinical and public health discourse, providing a pragmatic and policy-relevant language shift.

The prioritization of the research landscape permits the translation of knowledge into action. Policy recommendations include investing in upstream social interventions (such as protection of inclusive curricula, gender-affirming care protections, and housing zoning reforms), integrating SDOH into healthcare delivery (e.g., mandating screening and referral pathways, linking reimbursement to equity outcomes, and re-designing healthcare workforce education), expanding national research and data infrastructure (e.g., standardizing SDOH data collection methods, promoting data interoperability and integration among fields, and investing in robust evaluation procedures), and safeguarding equity-focused governance and workforce development (e.g., reinstating and expanding DEIA frameworks across federal, state, and local agencies, embedding SDOH competencies into accreditation processes, and investing in marginalized communities).

The shifting trajectory of Social Determinants of Health (SDOH) underscores a growing recognition that health outcomes are shaped more by social and structural contexts than by clinical care alone. From early empirical studies to today’s integrative frameworks and policy shifts, the understanding of SDOH has evolved into a dynamic, interdisciplinary field with the potential to transform health equity. However, this promise is challenged by shifting political climates, conceptual gaps, and implementation barriers. To sustain progress, it is imperative to reframe SDOH as relational and adaptable, invest in robust research infrastructure, and foster education and policies rooted in equity, resilience, and systemic change.

## Data Availability

No new data were created or analyzed in this study. Data sharing is not applicable to this article.
